# Supplementary material for: Early-exposure to new sex pheromone blends alters mate preference in female butterflies and in their offspring
Source: Nat Commun. 2020 Jan 2;11:53. doi: 10.1038/s41467-019-13801-2 (PMC6940390; doi:10.1038/s41467-019-13801-2)
Supplement: Supplementary file 1 — Supplementary Information [file 41467_2019_13801_MOESM1_ESM.pdf]

Early-exposure to new sex pheromone blend alters mate preference in female butterflies and in their offspring

Dion et al.

Supplementary Information

<u>Supplementary Methods 1</u>. MSP extraction and gas chromatography (GC) analyses of perfumed males.

Males were perfumed with 5µg of MSP2 diluted in hexane and were placed in pairs in a hanging cage, at the same temperature, humidity and light conditions as used for the mate choice experiments (see method section of the main document for description of the perfuming and the mating assay set-up). Total MSP from both hindwings and forewings were extracted from these males 30 minutes, 2 hours, 4 hours, 6 hours and 8 hours after perfuming. Some males were also coated with hexane only, and their MSP were extracted 30 minutes after perfuming. n=3 independent males per treatment were used. The extraction procedure, and the GC analysis, performed on a Shimadzu Gas chromatography-QQQ Mass Spectrometer, as well as the machine set-up, were described in Dion *et al*.[1]. Amounts of MSP2 were compared with a one-way ANOVA, followed by Turkey's pairwise comparisons and Bonferroni corrections in R v.3.2.4 [2] and RStudio v.1.0.136 [3].

<u>Supplementary Note 1</u>. Hexane did not impact naïve female mating outcome

Methods: To measure the impact of hexane on female innate mating bias, we performed mate choice assays where females were given a choice between a male that was perfumed with hexane (called 'Hex-male' below) and a wild type male that didn't receive any solvent (Wt-male). We used the same procedures as the ones detailed in the main method section. 2.5µL of hexane was applied to each hindwing androconia of the Hex-males. For both males to be submitted to the same amount of stress before the experiment, Wt-males were handled and manipulated exactly as Hex-males, except that they didn't receive any solvent. A black mark was applied with a sharpie pen randomly at the top or the bottom of their ventral hindwing in order to differentiate them. The two males were released in cylindrical hanging net cage and allowed to rest 30 minutes before the start of the mate choice assay. One naive female with its abdomen dusted with orange fluorescent powder was then introduced in the cage. Males were checked for presence of powder every 2 hours to prevent multiple mating. Assays were ended after 8 hours after the beginning of the experiment. All experiments were done at 24°C, 60% humidity, under UV and white light. Males and females were 4 and 3 days old, respectively, and the two males presented to each female for a mating trial had similar wing size and had dorsal forewing eyespot UV-reflective pupils. Similarity in mating outcomes was tested using a Pearson's χ^2 test using R v. 3.2.4 [2] implemented in RStudio v.1.0.136 [3], and blends were considered as altering female mating outcomes if these outcomes differed significantly from random mating (50:50).

Results: Out of 20 females, 11 mated with Hex-males, while 9 mated with Wt-males. Pearson χ^2 test confirmed a random mating, with absence of a female mating bias for either male type (χ^2 = 0.07; P value = 0.8). These results confirm that hexane doesn't impact female mating outcome.

(Source data are provided as a Source Data file).

(a)

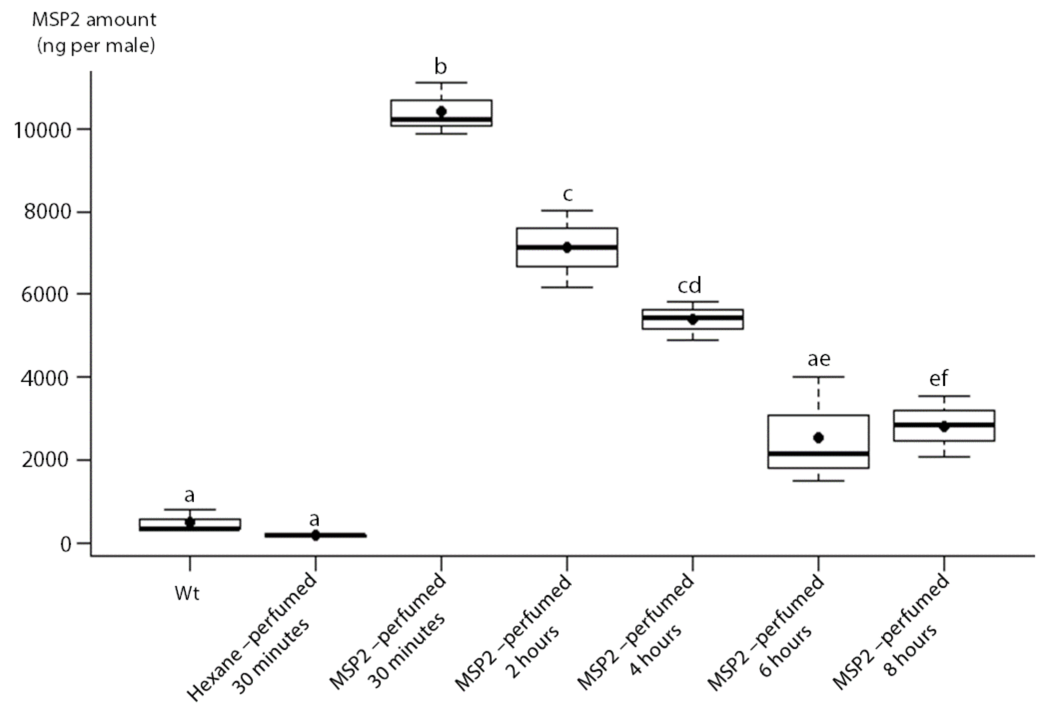

b)

P. values	Hexane - perfumed 30 minutes	MSP2-perfumed 30 minutes	MSP2-perfumed 2 hours	MSP2-perfumed 4 hours	MSP2-perfumed 6 hours	MSP2-perfumed 8 hours
MSP2-perfumed 30 minutes	3.40e-07	-	-	-	-	-
MSP2-perfumed 2 hours	2.10e-09	1.81e-03	-	-	-	-
MSP2-perfumed 4 hours	1.20e-05	0.26	1.80e-05	-	-	-
MSP2-perfumed 6 hours	0.03	5.50e-05	6.90e-08	7.30e-03	-	-
MSP2-perfumed 8 hours	0.01	1.1e-04	1.10e-07	1.76e-02	1	-
Wt	1.00	6.10e-07	3.20e-09	2.50e-05	4.81e-02	3.55e-02

Supplementary Figure 1. Hexadecanal amount of MSP2-perfumed males is higher than in Wt and hexane-perfumed males until 8 hours after treatment. (a) The horizontal lines and the points in each box are the median and the mean MSP2 amount, respectively. The 25th and 75th percentiles are contained within the outline of the boxes, and the horizontal lines above and below each box show the 1.5 times inter-quartile range of the data. Different letters above each box represent statistically different amounts, as shown by the P values calculated from pairwise comparisons after Bonferroni corrections (from ANOVA – see supplementary methods 1) (b). n=3 independent replicates per treatment. Source data are provided as a Source Data file.

Supplementary Table 1. Summary of the (a) reference tests, (b) GLMM outcomes (Figs. 2 and 3) and adjusted P values obtained from Tukey post-hoc tests (post GLMM) comparing the different treatments in experiment 1 (c) and experiment 2 (d).

**(a)**	**% female mating with NB male (total sample size)**	**Chi-squared**	**p-value**	**Conclusion**
Groups tested in experiment 1:				
Naïve females	7 (31)	15.16	9.89e-05	Mating bias for Wt blend
NB1-exposed females	15 (31)	0.03	0.86	No mating bias
Wt1-exposed females	3 (28)	20.16	7.12e-06	Mating bias for Wt blend
Offspring of NB1-exposed females	20 (46)	0.78	0.38	No mating bias
Offspring of Wt1-exposed females	14 (50)	9.68	1.86e-03	Mating bias for Wt blend
Groups tested in experiment 2:				
Naïve females	11 (37)	6.08	0.01	Mating bias for Wt blend
NB2-exposed females	31 (44)	7.36	6.66e-03	Mating bias for NB2
Wt2-exposed females	15 (29)	0.03	0.85	No mating bias
Offspring of NB2-exposed females	23 (46)	0.00	1.00	No mating bias
Offspring of Wt2-exposed females	22 (40)	0.40	0.53	No mating bias

**(b)** **GLMM Factors:**	**Statistics**	**P value**
Age of the male used for exposure (4, 5 or 6; analysis of the parental generation only)		
Experiment 1	χ^2_2=2.18	0.34
Experiment 2	χ^2_2=1.26	0.26
Age of the males used for mate choice (4, 5 or 6 day-old)		
Experiment 1	χ^2_2=3.73	0.15

Experiment 2	χ^2_2=2.25	0.32
Position of the black mark on the wing (top or bottom; experiment 2 only)		
Experiment 2	χ^2_1=1.32	0.25
Treatment (Naïve, Wt-exposed, NB-exposed, offspring of Wt-exposed and offspring of NB-exposed)		
Experiment 1	χ^2_4=15.14	4.44e-03
Experiment 2	χ^2_4=13.95	7.45e-03
Family		
Experiment 1	χ^2_1=0.44	0.50
Experiment 2	χ^2_1=0.00	1.00

**(c) Post-hoc tests for experiment 1**	Naïve females	Offspring of NB1-exposed females	Offspring of Wt1-exposed females	Wt1-exposed females
NB1-exposed females	0.48	1.00	0.68	1.87e-02
Wt1-exposed females	0.36	2.50e-02	0.20	
Offspring of Wt1-exposed females	0.99	0.71		
Offspring of NB1-exposed females	0.56			

**(d) Post-hoc tests for experiment 2**	Naïve females	Offspring of NB2-exposed females	Offspring of Wt2-exposed females	Wt2-exposed females
NB2-exposed females	3.70 e-03	0.28	0.59	0.49
Wt2-exposed females	0.38	0.99	0.99	
Offspring of Wt2-exposed females	0.18	0.99		
Offspring of NB2-exposed females	0.34			

Supplementary Table 2. Analysis of power of observed mating patterns in offspring of *B. anynana* females exposed to NB1 and Wt1-males

Proportion that mated with NB1 males (%)		Difference in proportion	Sample size used in the experiment		Total sample size required for α=0.05
Offspring of NB1-exposed females	Offspring of WT1-exposed females		Offspring of NB1-exposed females	Offspring of WT1-exposed females	
43	28	15	46	50	550

Power analysis done from a Fisher exact test, performed in G*Power v3.1.9.2 [4].

Supplementary References:

1 Dion, E., Monteiro, A. & Yew, J. Y. Phenotypic plasticity in sex pheromone production in *Bicyclus anynana* butterflies. *Sci. Rep.* **6**, 39002, doi:10.1038/srep39002 (2016).

2 R Development Core Team. *R: A language and environment for statistical computing*, <http://www.R-project.org> (2008).

3 RStudio Team. *RStudio: Integrated Development for R.* http://www.rstudio.com/, (2016).

4 Faul, F., Erdfelder, E., Lang, A.-G. & Buchner, A. G*Power 3: A flexible statistical power analysis program for the social, behavioral, and biomedical sciences. *Behavior Research Methods* **39**, 175-191 (2007).
