# Supplementary material for: Early-exposure to new sex pheromone blends alters mate preference in female butterflies and in their offspring
Source: Nat Commun. 2020 Jan 2;11:53. doi: 10.1038/s41467-019-13801-2 (PMC6940390; doi:10.1038/s41467-019-13801-2)
Supplement: Supplementary file 3 — Reporting Summary [file 41467_2019_13801_MOESM3_ESM.pdf]

nature research

Corresponding author(s): Dion Emilie

Last updated by author(s): Nov 11, 2019

Reporting Summary

Nature Research wishes to improve the reproducibility of the work that we publish. This form provides structure for consistency and transparency in reporting. For further information on Nature Research policies, see Authors & Referees and the Editorial Policy Checklist .

Statistics

For all statistical analyses, confirm that the following items are present in the figure legend, table legend, main text, or Methods section.

n/a	Confirmed	
☐	☒	The exact sample size (*n*) for each experimental group/condition, given as a discrete number and unit of measurement
☐	☒	A statement on whether measurements were taken from distinct samples or whether the same sample was measured repeatedly
☐	☒	The statistical test(s) used AND whether they are one- or two-sided<br>*Only common tests should be described solely by name; describe more complex techniques in the Methods section.*
☐	☒	A description of all covariates tested
☐	☒	A description of any assumptions or corrections, such as tests of normality and adjustment for multiple comparisons
☐	☒	A full description of the statistical parameters including central tendency (e.g. means) or other basic estimates (e.g. regression coefficient) AND variation (e.g. standard deviation) or associated estimates of uncertainty (e.g. confidence intervals)
☐	☒	For null hypothesis testing, the test statistic (e.g. *F*, *t*, *r*) with confidence intervals, effect sizes, degrees of freedom and *P* value noted<br>*Give P values as exact values whenever suitable.*
☒	☐	For Bayesian analysis, information on the choice of priors and Markov chain Monte Carlo settings
☒	☐	For hierarchical and complex designs, identification of the appropriate level for tests and full reporting of outcomes
☒	☐	Estimates of effect sizes (e.g. Cohen's *d*, Pearson's *r*), indicating how they were calculated

Our web collection on statistics for biologists contains articles on many of the points above.

Software and code

Policy information about availability of computer code

Data collection	No software was used for data collection
Data analysis	R v. 3.2.4 implemented in RStudio v.1.0.136, Packages lme4, multcomp, car, Rmisc and rcompanion.

For manuscripts utilizing custom algorithms or software that are central to the research but not yet described in published literature, software must be made available to editors/reviewers. We strongly encourage code deposition in a community repository (e.g. GitHub). See the Nature Research guidelines for submitting code & software for further information.

Data

Policy information about availability of data

All manuscripts must include a data availability statement. This statement should provide the following information, where applicable:

- Accession codes, unique identifiers, or web links for publicly available datasets
- A list of figures that have associated raw data
- A description of any restrictions on data availability

All data are available on the Institutional repository of the National University of Singapore ScholarBank@NUS (https://doi.org/10.25540/8Y6E-XPMY)

Field-specific reporting

Please select the one below that is the best fit for your research. If you are not sure, read the appropriate sections before making your selection.

☒ Life sciences ☐ Behavioural & social sciences ☐ Ecological, evolutionary & environmental sciences

For a reference copy of the document with all sections, see nature.com/documents/nr-reporting-summary-flat.pdf

Life sciences study design

All studies must disclose on these points even when the disclosure is negative.

Sample size	Sample size was established based on previous work in the same lab, where a sample size of ~25 was deemed to be sufficient to establish changes in a butterfly behavior due to learning (of wing patterns; Westerman et al. 2012, PNAS), and logistic constraints. No prior experiments had been done at this stage relative to adequate sample size to establish inheritance of a learned preference. This was the first study to do this in butterflies
Data exclusions	All females that have mated with both males and all females that have not mated at all within 6 hours were not included. This threshold was pre established using preliminary experiments. All details are in the manuscript and the suppl. mat.
Replication	This manuscript contains 2 replications of the same experiment but with different blends. Some results are similar, some different, all are discussed in the manuscript. A preliminary experiment, using a slightly different protocol (a natural exposure, instead of artificial exposure), also provided similar results.
Randomization	All individuals were isolated separately in containers prior to the experiment and allocated to the treatments randomly. Females from different treatment were tested for mating outcome at the same time, in different cages also allocated randomly and separated from each other by an opaque partition.
Blinding	All females remained isolated in containers and identified by a number with no indication of their treatment. The investigator performing the mating experiment had no indication of the female treatment and was often a different person than the one who performed the treatment prior to the mating experiment. Female treatment was assigned to the data only at the statistical analysis step.

Reporting for specific materials, systems and methods

We require information from authors about some types of materials, experimental systems and methods used in many studies. Here, indicate whether each material, system or method listed is relevant to your study. If you are not sure if a list item applies to your research, read the appropriate section before selecting a response.

Materials & experimental systems

n/a	Involved in the study
☒	☐ Antibodies
☒	☐ Eukaryotic cell lines
☒	☐ Palaeontology
☐	☒ Animals and other organisms
☒	☐ Human research participants
☒	☐ Clinical data

Methods

n/a	Involved in the study
☒	☐ ChIP-seq
☒	☐ Flow cytometry
☒	☐ MRI-based neuroimaging

Animals and other organisms

Policy information about studies involving animals; ARRIVE guidelines recommended for reporting animal research

Laboratory animals	It is unclear whether insects, for purposes of this question, are considered "animals". We used laboratory-reared wet season Bicyclus anynana butterflies for this study. Females were exposed at day 0, tested for mating outcome at day 2. Males were all from 4 to 6 days old.
Wild animals	The study did not involve wild animals
Field-collected samples	The study did not involve samples collected into the field
Ethics oversight	No ethical approval needed for work with wild type laboratory strains of insects

Note that full information on the approval of the study protocol must also be provided in the manuscript.
